# Editorial: Hybrids Part A: Hybrids for Tissue Regeneration

**DOI:** 10.3389/fbioe.2021.746641

**Published:** 2021-08-17

**Authors:** Kai Zheng, Antonio Jesus Salinas, Jonathan Lao

**Affiliations:** ^1^ Institute of Biomaterials, University of Erlangen-Nuremberg, Erlangen, Germany; ^2^ Departamento de Química en Ciencias Farmacéuticas, Instituto de Investigación Sanitaria Hospital 12 de Octubre, Universidad Complutense de Madrid, Madrid, Spain; ^3^ CIBER-BBN, Madrid, Spain; ^4^ Laboratoire de Physique de Clermont, Université Clermont Auvergne, Clermont-Ferrand, France

**Keywords:** wound healing, bone regeneration, scaffolds, hybrids, periodontal tissue regeneration

Numerous tissue engineering and regenerative medicine strategies have been applied in the past two decades to regenerate and reconstruct injured or diseased tissues. Many promising tissue regeneration strategies involve biomaterials (e.g., tissue engineering scaffolds, injectable formulations) (Gaharwar et al., 2020). Biomaterials can provide mechanical support and biochemical signals to encourage cell recruitment, attachment, growth, and differentiation and consequently guide tissue regeneration. Numerous organic (e.g., natural or synthetic polymers) or inorganic (e.g., bioceramics, bioactive glasses) materials have been successfully applied in various tissue regeneration scenarios (Gaharwar et al., 2020; Koons et al., 2020). Hybrids, commonly defined as consisting of components intimately mixed at the nanometer or molecular level—meaning that at least one of the components is circumscribed to areas ranging from several angstroms to a few hundred nanometers (Sanchez and Soler-Illia, 2006). Hybrids can be obtained by the homogeneous dispersion of nanoparticles inside a matrix or preferably by the entanglements or interpenetration of at least two different networks at the nanoscale—usually an organic one and an inorganic one. Since the nano dimension of the inorganic units and/or organic building blocks leads to more homogeneous materials and possible synergistic effects, hybrids exhibit unique physical, chemical, mechanical, and biological properties compared to their constituting phases. Hybrids have recently emerged as advanced biomaterials and are attracting increasing attention in tissue regeneration applications (Granel et al., 2019; Zhou et al., 2021).

This Research Topic entitled “*Hybrids for Tissue Regeneration*” shows the potential of hybrids in tissue regeneration. This topic invited researchers to contribute studies on recent advances in hybrid systems for tissue regeneration, including also (nano)composites since there are no clear borderlines between these materials (Kickelbick, 2006). We have collected one review article and nine original research articles, highlighting several emerging trends of hybrids and (nano)composites in the field of hard and soft tissue repair and regeneration.

Hybrids have shown impressive therapeutic effects in wound healing applications. Fu et al. reviews the recent development of metal-organic frameworks (MOFs) based hybrid systems as antibacterial agents, therapeutic delivery vehicles, and dressing systems for wound healing. They highlight the importance of surface engineering of MOFs with unique ligands in developing multifunctional hybrid systems. Cheng et al. developed freeze-dried hybrid dressings composed of epidermal growth factor and recombinant human-like collagen. Such a hybrid dressing significantly accelerated the healing of cutaneous wounds, as evidenced in a rat model. Yang et al. developed a hybrid delivery system composed of silk fibroin microparticles and insulin. This hybrid platform could release insulin in a sustained manner and consequently enhanced the healing of diabetic wounds.

Hybrids have also shown great potential in bone, cartilage, and periodontal tissue regeneration. Li et al. developed a gold nanoparticle/L-cysteine hybrid system to alleviate periodontal destruction by enhancing osteogenic differentiation of human periodontal ligament cells. Another important application of hybrids in implant dentistry, particularly in improving the resistance against bacterial infections around oral implants, was proposed by Wei et al. by using titanium reinforced with nanosized aggregates of graphene to enhance soft tissue sealing. Artificial ligaments for anterior cruciate ligament based on polyethylene terephthalate (PET) can also be improved by applying hybrid materials. Wu et al. show that PET artificial ligaments can be improved by coating them with antibacterial polydopamine and using this interlayer to introduce nano-hydroxyapatite and silver atoms to the PET surface, obtaining materials susceptible of further functionalization suitable for more advanced applications. In addition, Han et al. demonstrated that the cellular behavior of bone marrow mesenchymal stem cells (BMSCs), chondrocytes, and tendon stem cells could be modulated by controlling the pore size of melt electrowritten scaffolds based on polycaprolactone that is one of the most frequently used polymers for 3D printing. The results from Han et al. suggest that the biological properties of polycaprolactone-based hybrids and nanocomposites can be improved by modulating their morphology in addition to the composition optimization. Guided tissue regeneration (GTR) is a widely used clinical strategy for treating periodontal tissue defects. In this strategy, a membrane is applied to build a mechanical barrier from the gingival epithelium and hold space for periodontal regeneration. Zhou et al. (2021) developed fish collagen and polyvinyl alcohol (Col/PVA) dual-layer membranes for GTR. The collagen layer could enhance the osteogenic differentiation of BMSCs, while the PVA layer had the potential to prevent gingival epithelium ingrowth.

Hybrids based on bioceramics and bioactive glasses (BGs) are attractive biomaterials for hard tissue repair and regeneration, considering their bioactivity and osteogenic activity. Karbowniczek et al. successfully produced electrospun hybrid poly(3-hydroxybutyric acid-co-3-hydrovaleric acid) (PHBV)/HA fibrous scaffolds to biomimetic bone tissue. The combination of PHBV with HA significantly enhanced cell proliferation and filopodia formation responsible for cell anchoring within the created 3D environment. The obtained results show great potential in the development of hybrid scaffolds stimulating bone tissue regeneration. Han et al. developed BGs-modified hybrid dental resins. BGs enhanced the mechanical properties, biocompatibility, antibacterial and remineralizing activities due to the release of active ions from BGs. Hybridization with BGs or bioceramics provides an effective strategy to enhance the mineralization and osteogenic activity of polymeric matrices.

In summary, the Research Topic “*Hybrids Part A: Hybrids for Tissue Regeneration*” covers the recent development of hybrids for tissue regeneration. The editors hope this Research Topic will contribute to the progress in the field of hybrids for tissue regeneration and inspire researchers with sparkling ideas to further advance this field. Last but not least, we would like to thank all the authors, reviewers, and the development team for their efforts in producing this Research Topic.
